# Village chicken production and food security: a two-decade bibliometric analysis of global research trends

**DOI:** 10.1186/s40066-022-00379-0

**Published:** 2022-08-02

**Authors:** Emrobowansan Monday Idamokoro, Yiseyon Sunday Hosu

**Affiliations:** 1 Small-Scale Agribusiness and Rural Non-Farm Enterprise, Niche Area, Walter Sisulu University, P/Bag X1, Mthatha, 5117 South Africa; 2 Faculty of Commerce and Administration. Department of Economics and Business Sciences, Walter Sisulu University, P/Bag X1, Mthatha, 5117 South Africa

**Keywords:** Backyard chicken, Farming, Bibliometric evaluation, Food security, Vulnerable people

## Abstract

**Background:**

The present study aimed to reveal outputs of research works on village chicken production as a tool to combat food insecurity, taking into account the recurring challenge posed by food shortage and high rise in hunger among vulnerable people of several countries.

**Results:**

On aggregate, 104 publications were obtained in a BibTeX design for analysis using bibliometric package in R studio. The obtained data comprised, but not limited to authors, citations, institutions, key words and journals. Published articles on village chicken production with relation to food security retrieved from web of science (WOS) and Scopus data banks were utilized with a rise in research publications of a yearly growth of 12.93% during the study period. With regard to country, USA was ranked first with an aggregate sum of publications (*n* = 16), and a huge global academic influence with most top article citations (*n* = 509). The frequently used authors’ keywords in this studied research area were food security (*n* = 23), poultry (*n* = 9), chickens (*n* = 7), backyard poultry (*n* = 5), gender (*n* = 4), which all together created a hint on related studies on village chicken production and food security.

**Conclusions:**

The present study provides a worldwide situation that traverse the intellectual quandary on village chicken production and food security research, and a direction for further researches in this field. It is very vital to emphasize that the current study only dealt with principal areas of village chicken production as related to food security research, hence, it is projected that new empirical research and prospective research findings would afford new knowledge and insight on village chicken production as a means to address food security challenges as new studies evolves.

## Introduction

The quest for food by approximately ten (10) billion individuals in 2050 is projected to rise by 50% when placed side by side to 2012 [22]. At the moment, it is reported that over 820 million people are deprived of food with 2 billion suffering from micronutrient deficiency [62]. Likewise, the recent COVID-19 pandemic of 2019 has threatened to oppose years of progress on hunger and food deprivation by so many people [6]. It is believed by some quarters that the world have faced the worst economic recession from the last COVID-19 pandemic since the Great Depression era [63]. This situation invariable have left more people in lack of food and aggravated more hunger in several countries of the world.

With the current situation of hunger faced in certain parts of the world, achieving global food security will be one of the most critical challenges in the coming decades. Indeed, to date, the world has yet to come to terms with the international set goals for plummeting hunger within the periods of 1990 to 2015, i.e. lowering the numeric figure of hungry persons by 50% [18] and/or reducing by half the percentage of hungry individuals of the total global population [59]. Regardless of the vital progress that has been made in combating hunger, recent estimates show that about 805 million individuals were unable to get enough food in 2012–2014 [21]. Several hungry persons are known to live in developing countries and especially in Africa. According to FAO [21], it is reported that in some sub-Saharan African countries, there is a surge in the number of chronically hungry persons from around 176 million people in 1990–1992 to about 214 million in 2012–2014. There are variations in hunger levels among different groups/societies and this varies with different countries which basically are due to countless constraints related to political, economic, social, environmental variables and e interactions of these various variables. Despite the several challenges inherent in curbing hunger among millions of people in the world, the subject of finding ways to ensure that people are food secure cannot be over emphasized. One of the ways to combat the challenge of hunger and food insecurity among vulnerable people in most countries (especially developing countries) is to adopt a food production system that is sustainable, affordable and available to meet their nutritional needs. Food availability according to FAO [20] refers to foods of “appropriate nutritional quality”, and those which are socially and culturally acceptable by a group of people.

Village/small-scale/indigenous/backyard/family chicken production has been projected to be one of the easiest means of promoting food security by a large percentage of people globally [4, 7, 14, 21]. Village chicken production has been integrated with human livelihoods for thousands of years, enhancing diet, income, and food and nutrition security of the rural poor [4]. According to Wong et al. [63], village chicken is accessible to vulnerable groups of people, and it offer households with income and nutritionally rich food sources. Again, in an indirect manner, village chicken also improves food security in several ways such as improving nutrient utilization and recycling soil nutrients, adding to mixed farming practices, contributing to the empowerment of women, and assisting in getting access to healthcare and opportunity to education [63]. The potential contributions and impacts of village chicken/indigenous/backyard/small-scale scavenging poultry production systems in rural, resource-poor areas to several dimensions of food security in terms of availability, accessibility, utilization and stability have also been fully assessed [19, 49, 56]. Village chicken production is usually incorporated into mixed farming production systems with crops, and are a technique for vulnerable families to spread risks and stay food secured [56].

Village chicken like other livestock are known to produce meat (both muscle and organ meat) and eggs which constitute a high-quality food source, with vital macro- and micro-nutrients. Animal-source foods including chicken meat are particularly concentrated in highly bioavailable iron, several important vitamins and elements (such as vitamin A, vitamin B12, zinc, and riboflavin); which are nutrients that are often lacking in vegetarian foods observed in most vulnerable communities especially in developing countries [12, 13, 58]. Conversely, animal-source food including chicken with high concentrations of bioavailable nutrients is particularly essential for young children, pregnant and breast-feeding women who have high need of nutrient requirements, and elderly people who may have decreased intestinal absorption capacity [40]. Furthermore, income is often generated from the sale of village chicken and its products (e.g. egg) from both female-headed and male-headed households [2, 35]. Village chicken production is particularly an important income-generating venture for women, as they place little demand on mothers’ time, permitting suitable time allocation to child care, a vital element to achieving good nutrition [47].

Therefore, advancing the urge to promote scholarly research works on the production of village chicken to boost food security and lower the increasing rate of hunger around the globe is a worthwhile adventure. To the best of our knowledge, and based on the information that we can gather, there have been no published research work that have analysed scientific literatures on bibliometric studies on the current subject matter on village chicken production as a tool to boost global food security.

Furthermore, there are numerous growing research outputs done globally at an increased rate and it seems almost impossible to keep to pace with everything that is being published about a given subject matter at a glance. Hence, a precise study on important publication about a particular subject matter is of the essence. Assessing research articles on village chicken production as a prospective tool for boosting food security is very important with such studies being done with the use of bibliometric indexes which will further help to recognize hot, current and trending research topics, country contribution, international collaboration, and research inclinations on the subject matter. Therefore, the present study was carried out to assess and analyse research outputs on village chicken production as related to food security tool with the help of bibliometric instrument.

The technique commonly adopted to assess and analyse scientific work done on a specific subject matter is known as scientometrics or bibliometrics, which is different from systematic assessment and literature reviews. The chief aim of bibliometric/scientometric analysis is to evaluate trends of research, main research areas, top-cited articles in the field, local, national, and global influence, scientific contributions, and essential actors in a given research field. Numerous top-notch scientific research works including the reports of Khatun and Ahmed [28] and Ekundayo and Okoh [17] have been done using scientometric apparatuses to assess precise research done in different field of studies globally. Bibliometric evaluation allows people to utilize the instrumentality of both quantitative and qualitative analysis to measure and predict trends of scientific outputs and citation, largely focusing on published journal articles [43]. Qualitative bibliometric evaluation is regarded as an essential part in assessing the degree/extent of maturity of a research area [67].

The current study was sustained by a bibliometric analysis supported on existing data in the data banks retrieved for this investigation. Additional statistical, data mapping methods, mathematical calculations, and procedures were also included to improve the outcomes of this research work. Therefore, the chief motivation of this study is to analyse the worldwide trend of scientific research works on village chicken production as a useful tool for reducing hunger and food insecurity, by analytically conversing scholarly stands in the production of village chicken which presently is a growing interest globally and they are the two interconnecting research areas of the present study.

Conversely, the current research work highlighted some high flying categories on the utilization of village chicken in relation to food security studies, for instance, authors, research outputs, distribution of countries, the worldwide trends of citation, keywords, and trending topics on the subject matter.

The results from this study have a robust viewpoint of increasing the disciplinary knowledge bank of village chicken production as a viable tool for tackling hunger and food insecurity. By exploring outputs on scholarly research work, the present study will assist in recognizing potential areas of research gaps and research dynamics of village chicken production in relation to food security globally. Furthermore, we are optimistic that the study will also assist to advance and explore scientific researches done on village chicken production and propose on probable future research prospects.

## Materials and methods

### Descriptions of terms of analysed data

The bibliometrix analysis package that was utilized for the present research is an appropriate instrument known for precise package employed for data processing of articles, such as file conversion, descriptive analysis term extraction, similarity normalization for network analysis, duplicate matching and merging, and matrix building [5]. The data matrices employed were assembled from research publication dataset such as authors, words, countries, coupling, collaboration, multiple correspondence, keywords, and references for outputs like conceptual and co-citation framework analyses. The bibliometric graphical pairing/coupling occurs between two (2) research articles of say “*i* and *j*” that had their reference lists cited at least one common source [17]. The sum of bibliometric graphical pairing/coupling that results between research publications “*i* and *j*” or co-authorship in networking and scientific collaboration shows the strength of the network [5]. A particular collaboration/network signifies relationships in a system as a group of nodes and networks [66]. Conversely, the conceptual framework assessment made used of the K-means clustering and other measurement methods to characterize clusters of shared concepts known in bibliographic groupings. In addition, this K-means relies on word co-occurrences in a research publication dataset [5]. Scientific output or researchers’ contributions in a research area is analysed according on Lotka’s law. This law is an inverse square law which describes how often authors produce articles in their given area of specialty [31].

### Data processing and analysis

The present current study used relevant functions of bibliometrix R-package to evaluate retrieved data for the following descriptive result, citation analysis, authors’ scientific performance. Bibliometric scientific networks (e.g. citation, author keyword, and Keywords-Plus, author, nation links) and bibliometric graphical pairing/coupling (keyword co-occurrences and co-citation) were estimated and visualized from bibliometric bipartite (two-way) collaborations of rectangular yardsticks of research articles × attributes. For instance, the formula for a characteristic bibliometric network is given using this particular formula;

$${\text{Network}}\left( {\text{N}} \right) = {\text{X}} \times {\text{N}}^{{\text{T}}} ,$$

where X is a two-way (bipartite) collaboration/network matrix comprising research publications × attributes (e.g. countries, keywords, authors, and citations) and N is a symmetrical matrix ${\text{N = N}}^{{\text{T}}} {.}$

For the current study, a graphical model was used for all collaborations by employing the force-directed Fruchterman algorithms applied in the networkPlot command of the bibliometrix R-package. Subsequently, all the networks/links were standardized by using the Salton’s cosine coefficient, proximity indices/matrixes (association strength/power), Simpson’s coefficient (inclusion indices), and the Jaccard’s similarity indices among nodes of a network/collaboration [5]. Furthermore, the k-means clustering was done on keywords to assess concepts in village chicken production as a significant food security tool by utilizing the function of conceptual framework of the bibliometrix R-package. This function employed the Porter’s stemming algorithm [45] to regulate adjusted words to their exact form.

### Retrieval of data used for analysis

The current study employed the utilization of scientific outputs which were published on village chicken in relation to food security research outputs. These data were obtained from the combination of Scopus and Web of Science (WOS) archive on 15 August, 2021. Web of Science and Scopus data banks hosted reliable and efficient high-impact scientific publications [32, 41, 48]. Therefore, the authors made use of Scopus and WOS to collect data for the intended objective. It should be noted that we used the advanced search function in Web of Science and Scopus as a result of the fact that both data banks allow for building long and combined search queries. Ordinarily, in research publications that involve bibliometric study, one database can be used reason being that bibliometric indicators and literature mapping are difficult to carry out on publications retrieved from more than one data bank [53, 54]. Conversely, it has been reported that the use of a single data bank for information retrieval during bibliometric studies may exclude some vital publication on a particular subject matter which may be required for analysis on that subject matter [41]. The use of Scopus and WOS data banks will therefore guarantee 100% inclusion of PubMed articles. Therefore, Scopus and WOS are guaranteed to have an all-inclusive collection of articles in PubMed and other scientific data bases.

### Search strategy utilized for collection of data

In order to effectively create a search query that is suitable to collect most of the related volume of research articles with slightest false-positive out-come, we carried out an exhaustive literature review research on the intended subject matter, and chiefly on studies and systematic reviews to accustom ourselves with most of the possible keywords associated to the searched subject matter. The searched method strategy for data collection that we employed has also being used by other authors [23, 29, 33, 36]. The simple procedure that we used in the search for data assembling was to use the title/abstract search methodology for keywords related to “village chicken” and “food security”. However, employing such kind strategy could result in the retrieval of a large number of publications that may be irrelevant for the purpose of our study. Therefore, in order to narrow-down the title/abstract method that we utilized, a particular constraint was adopted which included the presence of certain “terms” associated to village chicken or food security in addition to the title/ abstract strategy.

### Search query employed for data collection for this study

The search query used for the present study comprised specific phrases related to village chicken with specific phrases associated to food security which were entered into the title/abstract search engine. This was followed by some precise terms as a limit to lessen and eliminate unwanted research outputs that are not originally a part of the objective of the present study. The search queries that we employed for Web of Science (WOS) and Scopus are given below;

#### WOS

114 results from Web of Science Core Collection for:

Food security* AND (backyard chicken* OR village chicken* OR local chicken* OR indigenous chicken*) (All Fields).

Refined By: Search within all fields: (Women* Or Feather* Or Egg* Or Children* Or Poor* Or Rural* Or Poverty Alleviation* Or Nutrition* Or Income* Or Food*).

#### SCOPUS

19 document results.

(TITLE-ABS-KEY (food AND security* AND (backyard AND chicken* OR village AND chicken* OR local AND chicken* OR indigenous AND chicken*))) AND ((women* OR feather* OR egg* OR children* OR poor* OR rural* OR poverty AND alleviation* OR nutrition* OR income* OR food*)).

### Data analysis and processing

**Fig. 1:**
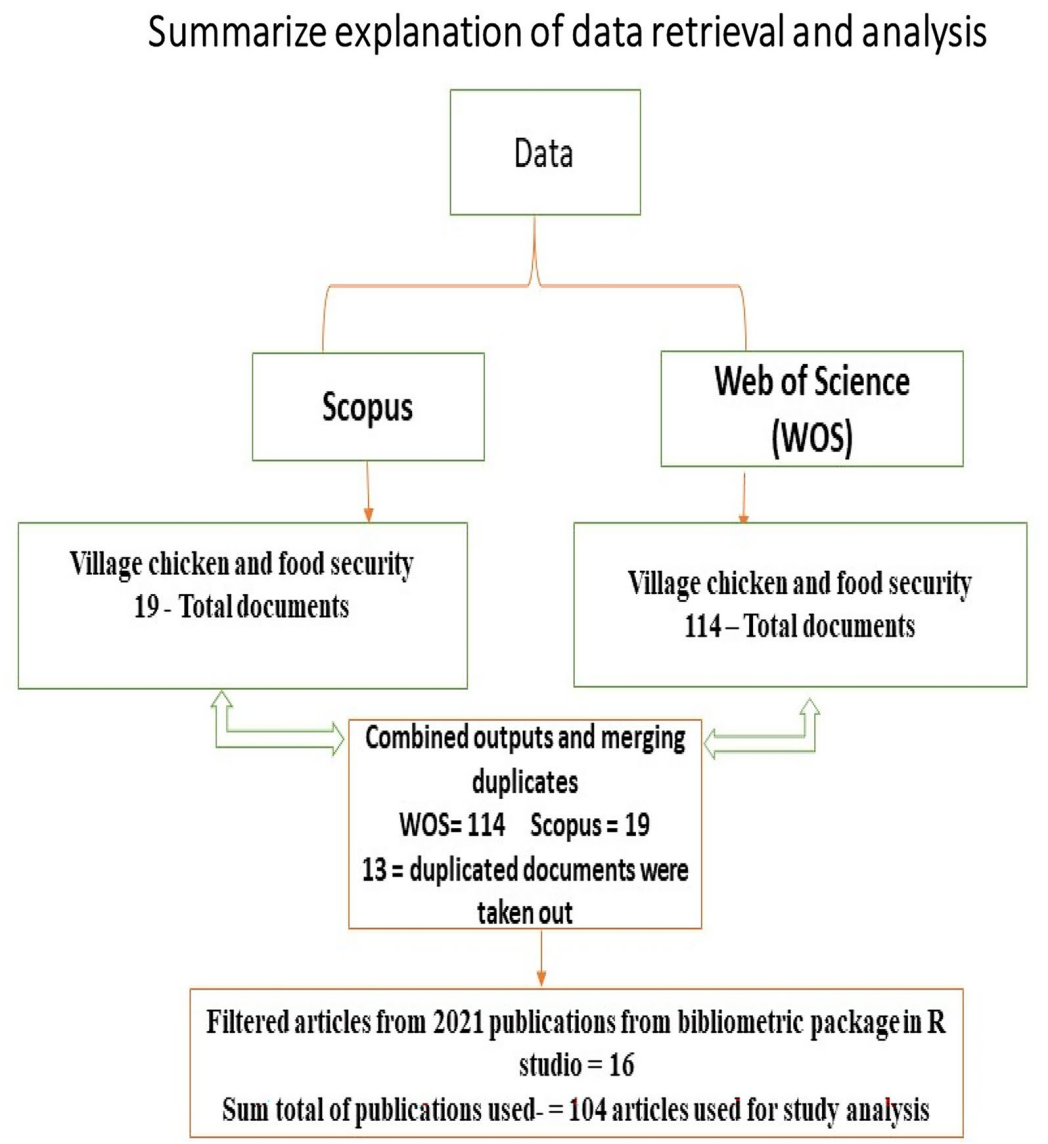
Pictorial explanation depicting the inclusion and exclusion criteria for articles selection

The present study analysed all retrieved data from Scopus and WOS data bank by utilizing RStudio v.4.0.4 software with bibliometrix R-package for scientometric features [5]. All data obtained were then transferred into R Studio and refined into a bibliographic data format before they were organized to take out duplications that may arise from the two data banks (Scopus and WOS) that we used. A pictorial representation of the retrieval and analysis of data is explained in Fig. 1. Furthermore, for us to evade article duplication extracted from the two data banks, all replicated peer-reviewed articles were restricted to one record in the analysis. For visualization purpose, the names of authors, author’s keywords (DE), and keywords-plus (ID) were extracted to better understand the knowledge structure of this particular subject matter (village chicken). All retrieved data were reviewed for variant names, spelling errors and associations. For keywords (DE) and keywords-plus (ID), the subject of the current study (village chicken and food security) was given a principal term to terms. Furthermore, the co-occurrence of a phrase in the keywords (DE set) and keywords-plus (ID set) of authors in the dataset was assessed as a group made of the two sets (DE and ID) that converge. The VOSviewer software (www.vosviewer.com, version 1.6.17) was employed to obtain a visual representation of collaborations between countries and authors using network maps [60]. Similarity processes and set-based test were analysed using a VOSviewer for the keywords or a similar keyword in an article which contributed to research on village chicken and food security.

## Results

**Fig. 2:**
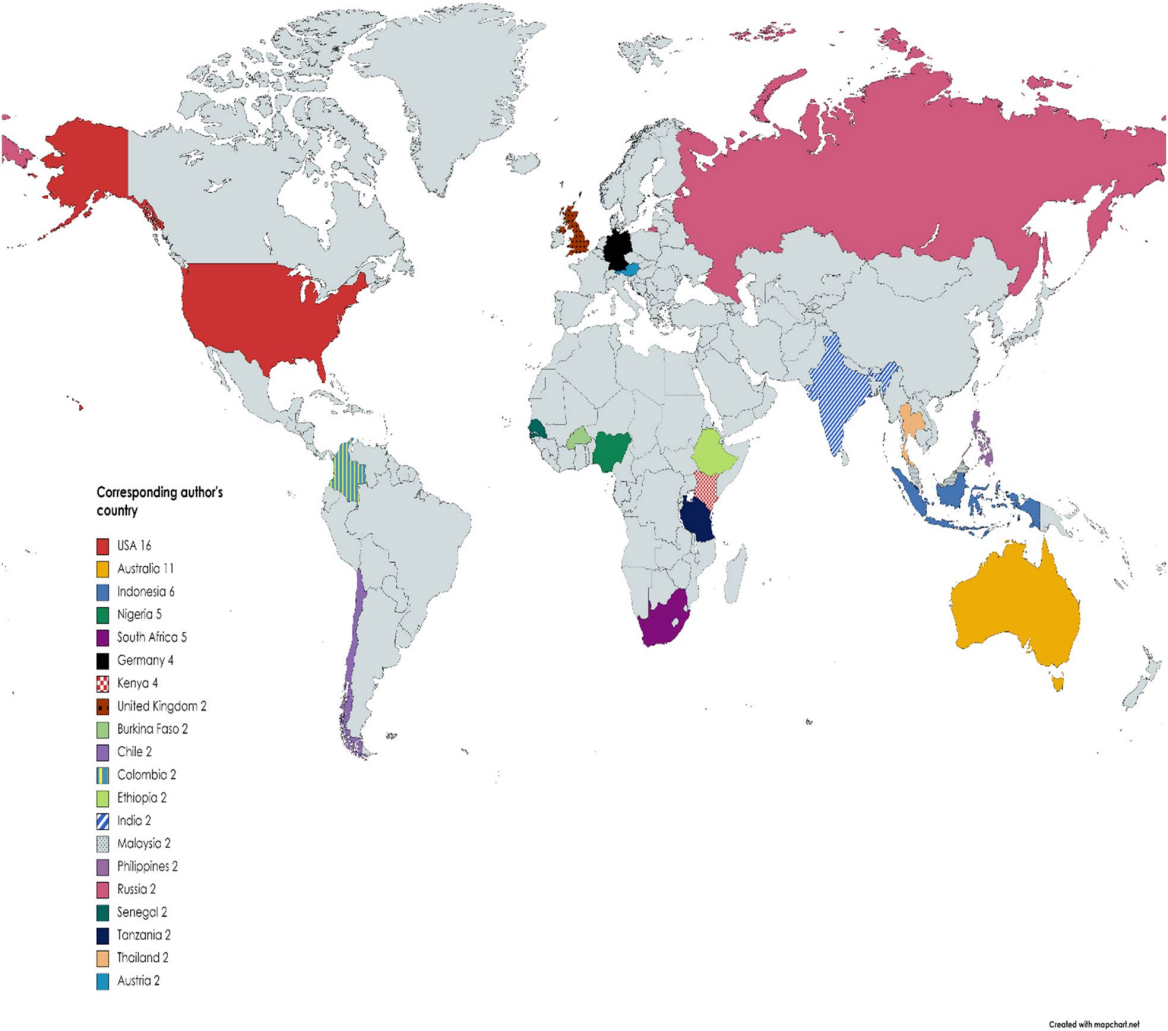
Spatial mapping of the top 20 most productive nations based on number of research articles on village chicken production and food security studies (corresponding author’s countries). Grey colour areas depict the zones that are not among the top 20 countries

From the present study, an aggregate of 104 publications which were published from the year 2000 to 2020 of the study time, with the analysis characteristics shown in Table 1. The outcomes for the studied periods composes of 446 authors, with 11 single author, 0.233 article per author (4.29 authors per article), a collaboration index of 4.69 and a 4.7 co-authors per article. Apart from the 11 single authors, all the rest authors (436) had a multi-author study outputs. In addition an aggregate of 12.02 citations per article was archived during from the present study. Figure 2 shows research articles of worldwide mappings related with village chicken and food security research for the peak 20 topmost active countries (established based on corresponding authors’ countries). Judging from the result in Fig. 2, USA ranked number one in the total sum of articles (*n* = 16), followed by Australia (*n* = 11), Indonesia (*n* = 6), Nigeria (*n* = 5) and South Africa (*n* = 5), respectively, among other countries.

**Table 1:** Summarized information of recovered published documents on village chicken production and food security from WOS and Scopus data bases

Main information about data	Results
Timespan	2000:2020
Sources (journals, books, etc.)	77
Documents	104
Average years from publication	5.16
Average citations per documents	12.02
Average citations per year per doc	1.67
References	247
Document types	
Article	80
Article; book chapter	3
Article; proceedings paper	4
Book chapter	1
Editorial material	1
Proceedings paper	10
Review	5
Document contents	
Keywords plus (ID)	508
Author’s keywords (DE)	386
Authors	
Authors	446
Author appearances	489
Authors of single-authored documents	11
Authors of multi-authored documents	436
Authors collaboration	
Single-authored documents	11
Documents per author	0.233
Authors per document	4.29
Co-Authors per documents	4.7
Collaboration index	4.69

**Fig. 3:**
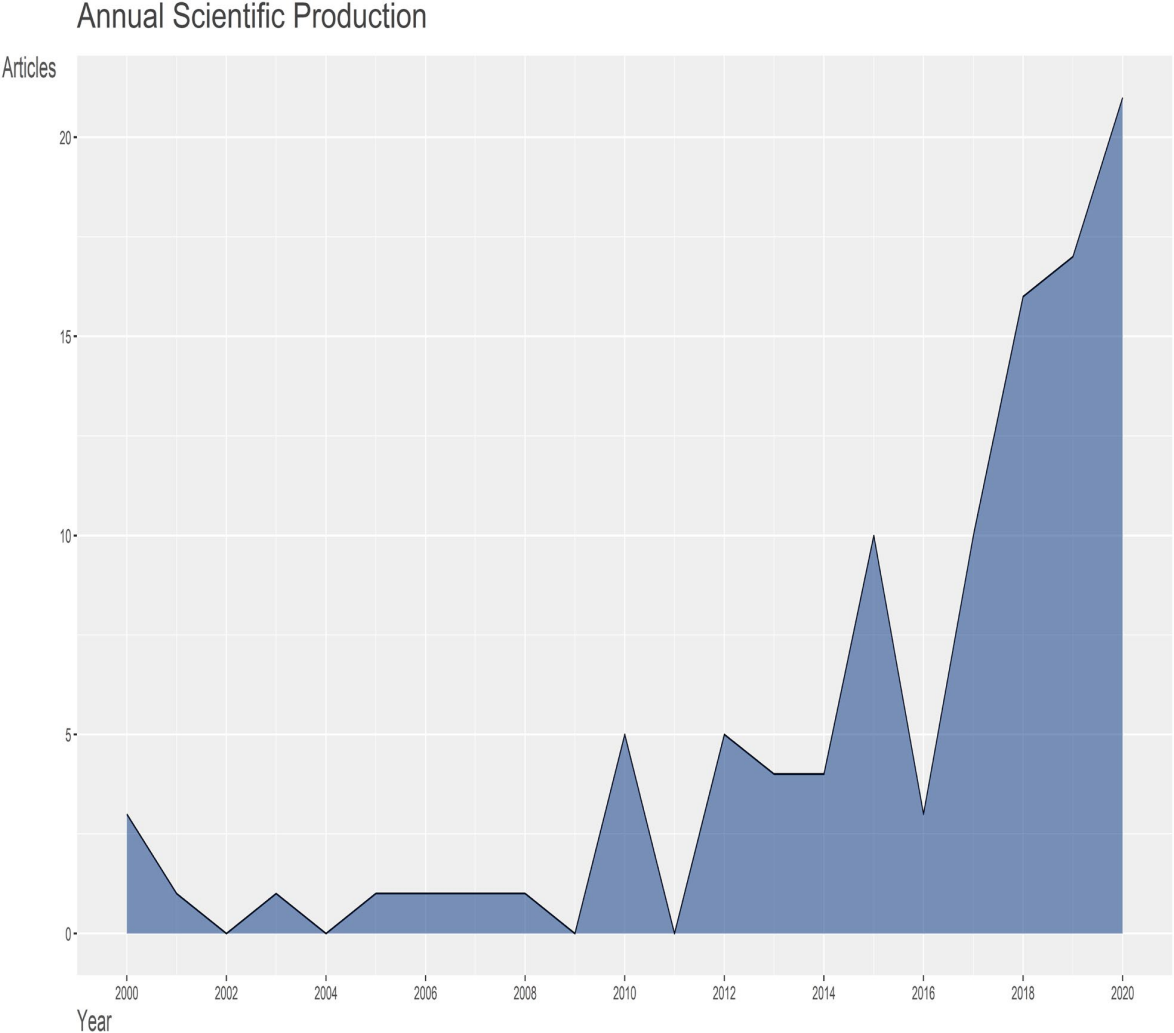
Inclinations of annual scientific publications (from 2000 to 2020) in village chicken and food security research with an annual growth rate of 12.93%. Village chicken and food security research studies showed obvious fluctuations (downward and upward trends) in research articles between 2002 and 2016 then a steady increase after then

Likewise, in Fig. 3, our result showed a pattern in research outcomes on village chicken with regard to food security with a yearly growth rate of 12.93%. It could be seen from Fig. 3 that there were fluctuations with a downward and upward trends in the total publications between 2002 and 2016. However, the trend then gained a steady upward trend after 2016 (Fig. 3). The year with the highest number of article publications in village chicken with regard to food security research field was in the year 2020 with an aggregate of 21 articles (Fig. 3). The result for the average article citations (AAC) of countries with most cited publications in the field of village chicken production and food security research showed that Belgium (55), Colombia (32), USA (31.8) and France (29) leads the chart, respectively (Table 2). The research publications associated with village chicken farming and food security work for the top 20 most productive nations are presented in Table 3. Among these top nations, USA is ranked first in terms of the aggregate number of research outputs (*n* = 16), followed by Australia (*n* = 11), Indonesia (*n* = 6), Nigeria (*n* = 5) and South Africa (*n* = 5), respectively. The frequency of research articles varied among the top 20 nations from 0.0101 to 0.1616.

**Table 2:** The topmost 20 most cited nations in relation to the average article citations (AAC) in the field of village chicken production and food security from 2000 to 2020

S/N	Nations	Total citations	Average article citations
1	USA	509	31.8
2	Australia	135	12.3
3	Kenya	85	21.2
4	Indonesia	78	13
5	Colombia	64	32
6	Belgium	55	55
7	Germany	41	10.20
8	United kingdom	34	8.50
9	France	29	29
10	Ethiopia	24	12
11	Senegal	22	11
12	Portugal	19	19
13	South Africa	17	3.40
14	Mozambique	16	16
15	Nigeria	15	3
16	Chile	13	6.50
17	Malaysia	12	6
17	Niger	11	11
19	Denmark	6	6
20	Ireland	6	6

**Table 3:** The top 20 publications by nations on research in the field of village chicken production and food security

S/N	Country	Position	Articles	Frequency	SCP	MCP	MCP_ratio
1	USA	1	16	0.1616	4	12	0.75
2	Australia	2	11	0.1111	3	8	0.72
3	Indonesia	3	6	0.0606	5	1	0.16
4	Nigeria	4	5	0.0505	4	1	0.20
5	South Africa	4	5	0.0505	5	0	0
6	Germany	5	4	0.0404	1	3	0.75
7	Kenya	5	4	0.0404	2	2	0.50
8	United kingdom	5	4	0.0404	0	4	1
9	Burkina Faso	6	2	0.0202	0	2	1
10	Chile	6	2	0.0202	2	0	0
11	Colombia	6	2	0.0202	0	2	1
12	Ethiopia	6	2	0.0202	2	0	0
13	India	6	2	0.0202	2	0	0
14	Malaysia	6	2	0.0202	2	0	0
15	Philippines	6	2	0.0202	1	1	0.50
16	Russia	6	2	0.0202	2	0	0
17	Senegal	6	2	0.0202	1	1	0.50
18	Tanzania	6	2	0.0202	1	1	0.50
19	Thailand	6	2	0.0202	1	1	0.50
20	Austria	7	1	0.0101	1	0	0

*SCP* single country publications, *MCP* multiple country publications

In addition, the countries that occupied the topmost position in terms of multiple country publications (MCP) and networking were; the USA which is ranked in first place (*n* = 12), while Australia (*n* = 8), United Kingdom (*n* = 4) and Germany (*n* = 3) followed in the order of position, respectively. Meanwhile, the countries ranked in top positions for single country publications (SCP) of research articles in the field of village chicken farming and food security research are Indonesia and South Africa (*n* = 5) in the first positions while USA and Nigeria (*n* = 4) were both placed in the second position, respectively (Table 3). Judging from the result in Table 4, the most important author keywords in the research field of village chicken and food security publications are, the keyword such as food security (*n* = 23) was ranked first, followed by, poultry (*n* = 9) and chickens (*n* = 7) among others.

**Table 4:** Most relevant words used by authors in the field of village chicken production and food security research

S/N	Author keywords (DE)	Occurrences	Keywords plus (ID)	Occurrences
1	Food security	23	Food security	13
2	Agriculture	4	Chicken	15
3	Newcastle disease	7	Quality	7
4	Nutrition	5	Animal	5
5	Poultry	9	Poultry	9
6	Livestock	4	Family poultry	8
7	Chicken	11	Newcastle disease	5
8	Gender	4	Health	7
9	Backyard poultry	5	Security	6
10	Kenya	4	Impacts	6

**Fig. 4:**
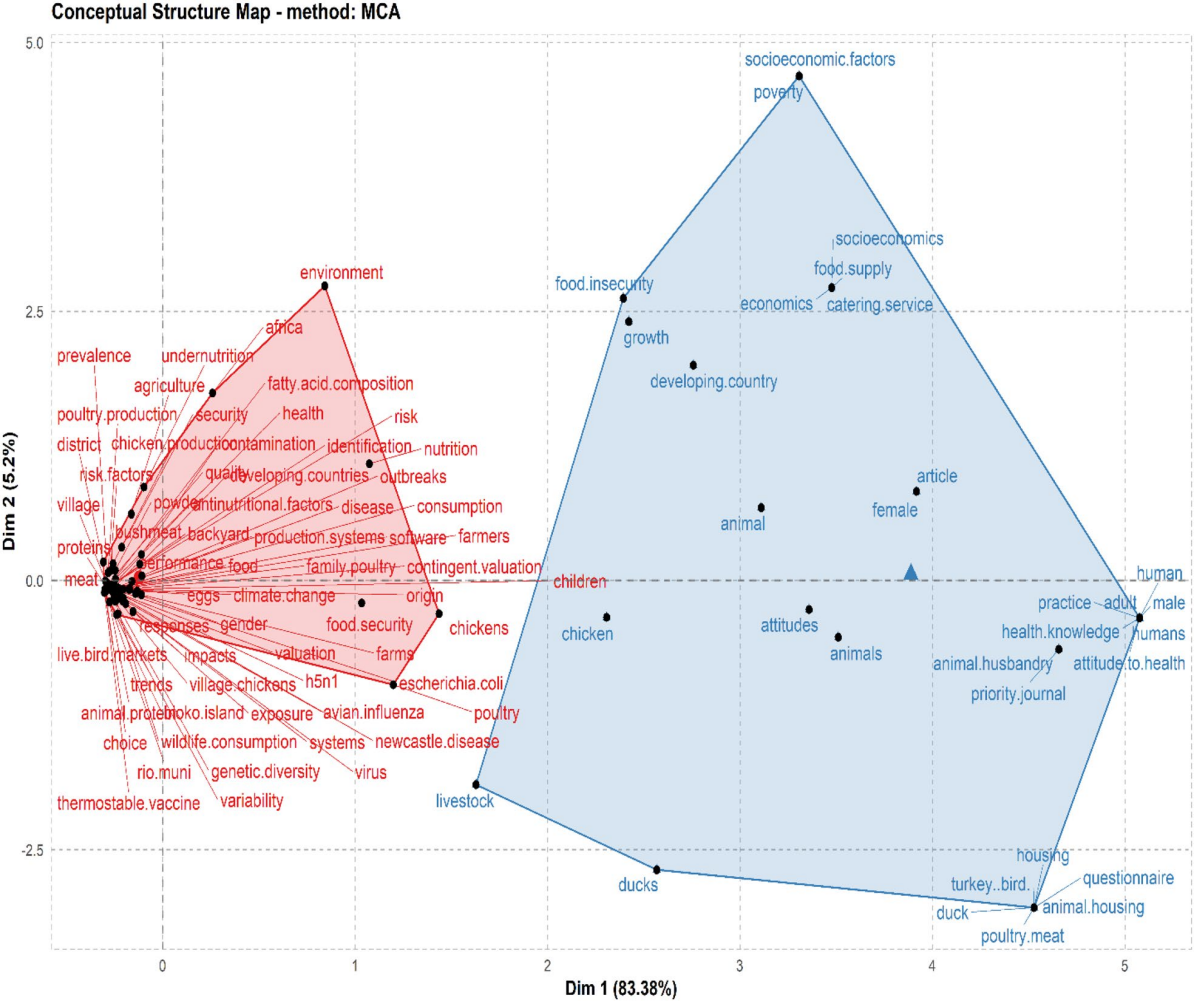
Common conceptual frames related with village chicken production and food security research studies. The 104 selected publications indicated K-means clustering with two (2) clusters reflecting concepts of village chicken (including birds, poultry, chickens, nutrition, food, performance, health, agriculture, family poultry, consumption, farmers) for boosting food security (e.g. socio-economics, food supply, food insecurity, catering service, human, female, health-knowledge, attitudes, attitude to health, etc.) which are commonly linked to village chicken farming and food security studies

In Table 5, the top 20 most relevant authors in the field of village chicken production in relation to food security were presented with the first author by the name R, Alders occupying the first position (*n* = 6) based on the number of publications that he has produced. However, W Maulaga who is another author was ranked second (*n* = 4), followed by O, Adebambo and B.J., De (*n* = 3) who were placed in the third position, respectively. With regard to total citations, the H_index for R, Alders was 5 (TC = 103) who maintained the first position. However, the author by name W, Maulaga was in the second position with H_index of 4 (TC = 45). The author who clinched the third position for top ranked H_index was B.J., De with H_index of 3 (TC = 74). The shared conceptual frames of retrieved publications as was explained by K-means clustering with two (2) clusters reflecting concepts of village chicken production (including poultry, chickens, birds, nutrition, food, performance, health, agriculture, family poultry, consumption, farmers) for boosting food security (e.g. socio-economics, food supply, food insecurity, catering service, human, female, health-knowledge, attitudes, attitude to health, etc.) which are commonly linked to village chicken farming and food security studies (Fig. 4).

**Table 5:** Top 20 relevant authors on village chicken production and food security research

S/N	Author	Position	H_index	G_index	M_index	TC	NP	% of 104	PY_start
1	Alders R	1	5	6	1	103	6	5.76	2017
2	Maulaga W	2	4	4	0.80	45	4	3.84	2017
3	Adebambo O	3	1	2	0.10	6	3	2.88	2012
4	De B J	3	3	3	0.60	74	3	2.88	2017
5	Akoko J	4	2	2	0.40	27	2	1.92	2017
6	Alarcon P	4	2	2	0.40	27	2	1.92	2017
7	Bagnol B	4	2	2	0.40	62	2	1.92	2017
8	Campbell Z	4	2	2	0.50	22	2	1.92	2018
9	Cardona C	4	2	2	0.16	14	2	1.92	2010
10	Costa R	4	2	2	0.50	19	2	1.92	2018
11	Coulibaly K	4	1	2	0.25	4	2	1.92	2018
12	Dessie T	4	2	2	0.28	14	2	1.92	2015
13	Dumas S	4	2	2	0.33	35	2	1.92	2016
14	Galdames P	4	2	2	0.66	13	2	1.92	2019
15	Gallardo R	4	1	1	0.25	3	2	1.92	2018
16	Garikipati S	4	2	2	0.28	14	2	1.92	2015
17	Gueye E	4	2	2	0.09	38	2	1.92	2000
18	Hamilton-West C	4	2	2	0.66	13	2	1.92	2019
19	Kenis M	4	1	2	0.25	4	2	1.92	2018
20	Marsh T	4	2	2	0.50	22	2	1.92	2018

Ranking is based on number of publication (NP)

*NB* % of 104 total sum of articles published on village chicken production and food security between 2000 and 2020, *NP* number of publications, *PY_start* publication year start

Table 6 shows the top 20 most cited articles on village chicken production and food security based on total citations from 2000 to 2020. The article written by G.C. Canonico, (2005) was ranked in the first position with a total citation of 138. The article that was ranked in the second position was authored by K.W. Lee (2010) with an aggregate citation of 78. The third and fourth position had N, Van Vliet (2015) and Foerster (2012) with total citations of 56 and 55, respectively (Table 6). In addition, the top 20 journals with the most published articles in the field of village chicken production and food security listed in Table 7. These journals include; Tropical Animal Health and Production, Food Security, Plos one and Asian-Australasian Journal of Animal Sciences among others. However, the journal of Tropical Animal Health and Production ranked first (Number of publication = 6; H_index = 4) among the journals with most published articles. This was followed by Food Security Journal (Number of publication = 4; H_index = 3). Plos One journal was also ranked in the second position with number of publications (4 each) and H_index (3). Conversely, Asian-Australasian Journal of Animal Sciences journal was ranked in the third position among the journals with most published articles with total publication of 3 and H_index of 2 (Table 7).

**Table 6:** Top 20 most cited articles on village chicken production and food security research from 2000 to 2020

S/N	First author and year	Journal name	DOI	Total citations	TC per year	Normalized TC
1	Canonico GC, 2005	Aquatic Conservation: Marine and Freshwater Ecosystems	10.1002/aqc.699	242	14.23	1
2	Lee KW, 2010	Poultry Science	10.3382/ps.2009–00,418	138	11.50	4.08
3	Van Vliet N, 2015	Ethnobiology and Conservation	NA	78	11.14	4.35
4	Foerster S, 2012	Conservation Biology	10.1111/j.1523-1739.2011.01802.x	56	5.60	2.24
5	Wong JT, 2017	Global Food Security- Agric Policy Journal	10.1016/j.gfs.2017.04.003	55	11	4.86
6	Gilbert M, 2015	Plos One	10.1371/journal.pone.0133381	55	7.85	3.07
7	Ayieko MA, 2016	Journal of Insects as Food and Feed	10.3920/JIFF2015.0080	42	7	1.90
8	Martens SD, 2012	Journal of Agriculture and Rural Development in the Tropics and Subtropics	NA	42	4.20	1.68
9	Paul M, 2013	Acta Tropica	10.1016/j.actatropica.2013.01.008	29	3.22	2.32
10	Gueye EH, 2000	Development in Practice	10.1080/09614520052565	25	1.13	2.58
11	Van Vliet N, 2015	Oryx	10.1017/S0030605313000549	22	3.14	1.22
12	Maass BL, 2012	Tropical Animal Health and Production	10.1007/s11250-011-0061-5	21	2.1	0.84
13	Gebremedhin S, 2017	Nutrition	10.1016/j.nut.2016.06.002	21	4.20	1.85
14	Ward JD, 2014	Food Security	10.1007/s12571-014-0374-0	20	2.50	2.28
15	Dumas SE, 2016	Food Security	10.1007/s12571-016-0579-5	19	3.16	0.86
16	Lipoeto NI, 2001	Asia Pacific Journal of Clinical Nutrition	10.1046/j.1440-6047.2001.00201.x	19	0.90	1
17	De Almeida AM, 2008	Tropical Animal Health and Production	10.1007/s11250-008-9130-9	19	1.35	1
18	Bett HK, 2013	Food Policy	10.1016/j.foodpol.2013.05.012	18	2	1.44
19	Alders RG, 2018	Maternal and Child Nutrition	10.1111/mcn.12668	16	4	2.41
20	Onono JO, 2018	Agricultural Systems	10.1016/j.agsy.2017.10.001	14	3.50	2.11

**Table 7:** The topmost 20 publications that are relevant in the field of village chicken production and food security research from 2000 to 2020

S/N	Source	H_index	G_index	M_index	TC	NP	PY_start
1	Tropical Animal Health and Production	4	6	0.285714286	54	6	2008
2	Food Security	3	4	0.375	53	4	2014
3	Plos one	3	4	0.428571429	82	4	2015
4	Asian-Australasian Journal of Animal Sciences	2	2	0.090909091	5	3	2000
5	International Conference: Improving Tropical Animal Production for Food Security	0	0	0	0	3	2020
6	Maternal and Child Nutrition	3	3	0.75	35	3	2018
7	Preventive Veterinary Medicine	3	3	0.75	26	3	2018
8	2nd International Conference on Food Security and Sustainable Agriculture in the Tropics	0	0	0	0	2	2020
9	Agricultural Systems	2	2	0.5	16	2	2018
10	Avian Diseases	2	2	0.166666667	5	2	2010
11	Frontiers in Veterinary Science	2	2	0.4	18	2	2017
12	Global Food Security-Agriculture Policy Economics and Environment	1	2	0.2	55	2	2017
13	Journal of Insects as Food and Feed	2	2	0.333333333	45	2	2016
14	Vaccine	2	2	0.285714286	15	2	2015
15	Worlds Poultry Science Journal	2	2	0.2	14	2	2012
16	2019 IEEE 5th International Conference for Convergence in Technology (I2CT)	0	0	0	0	1	2019
17	Acta Tropica	1	1	0.111111111	29	1	2013
18	African Journal of Biotechnology	1	1	0.083333333	9	1	2010
19	African Journal of Science Technology Innovation\& Development	0	0	0	0	1	2018
20	Agricultural Economics	1	1	0.333333333	3	1	2019

*NP* number of publication, *TC* total citations, *PY_start* publication year start

**Fig. 5:**
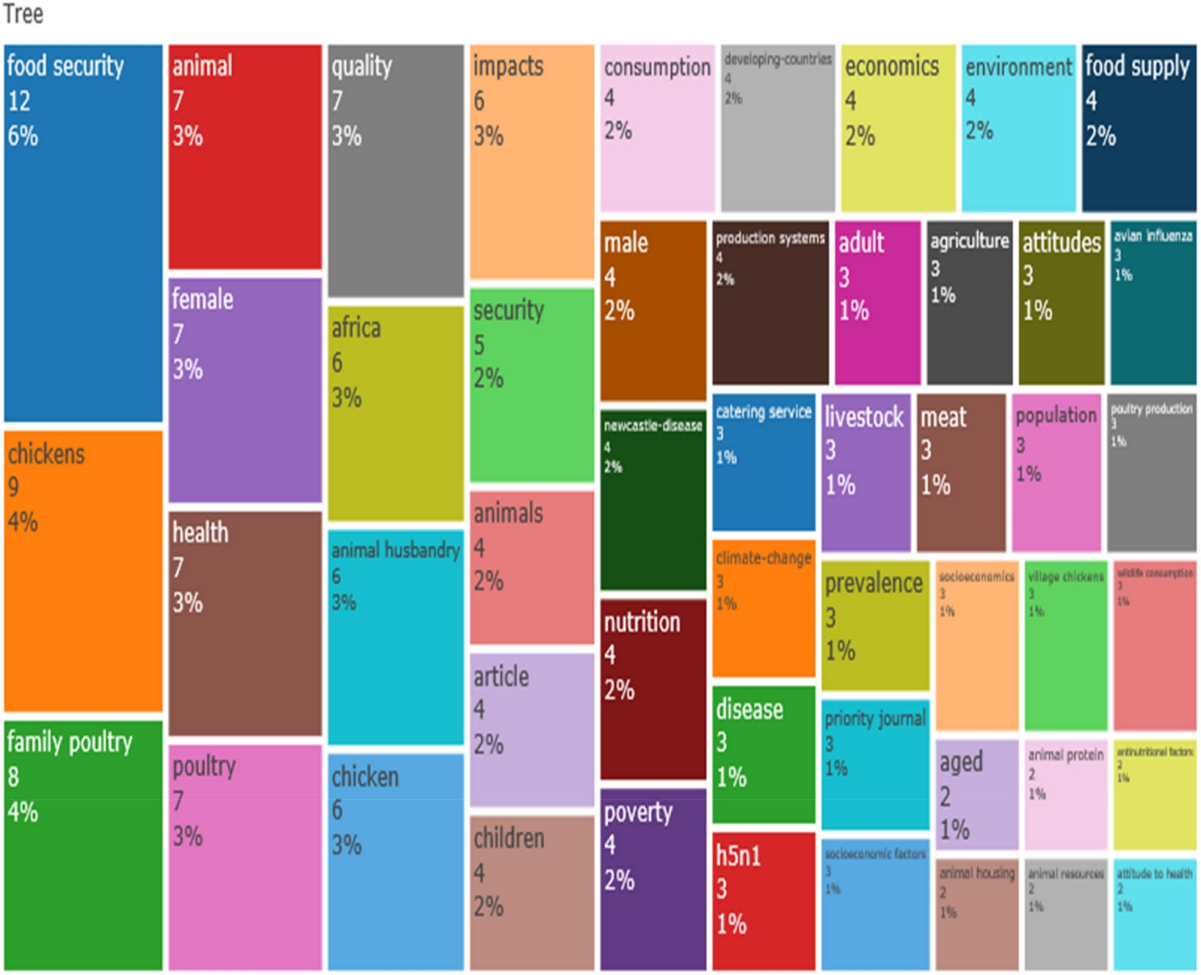
Tree map of discipline distribution in the field of village chicken production and food security research.

From statistical evaluation of the articles related to the production of village chicken in relation to food security, we can deduce that village chicken production as a means of reducing food insecurity involves several research directions, including nutrition, animal husbandry, health, economics and environmental science and climate change, among others. The current study presents a better motivation for the advancement and a large space for research in the field of village chicken production in curbing food insecurity (Fig. 5).

**Fig. 6:**
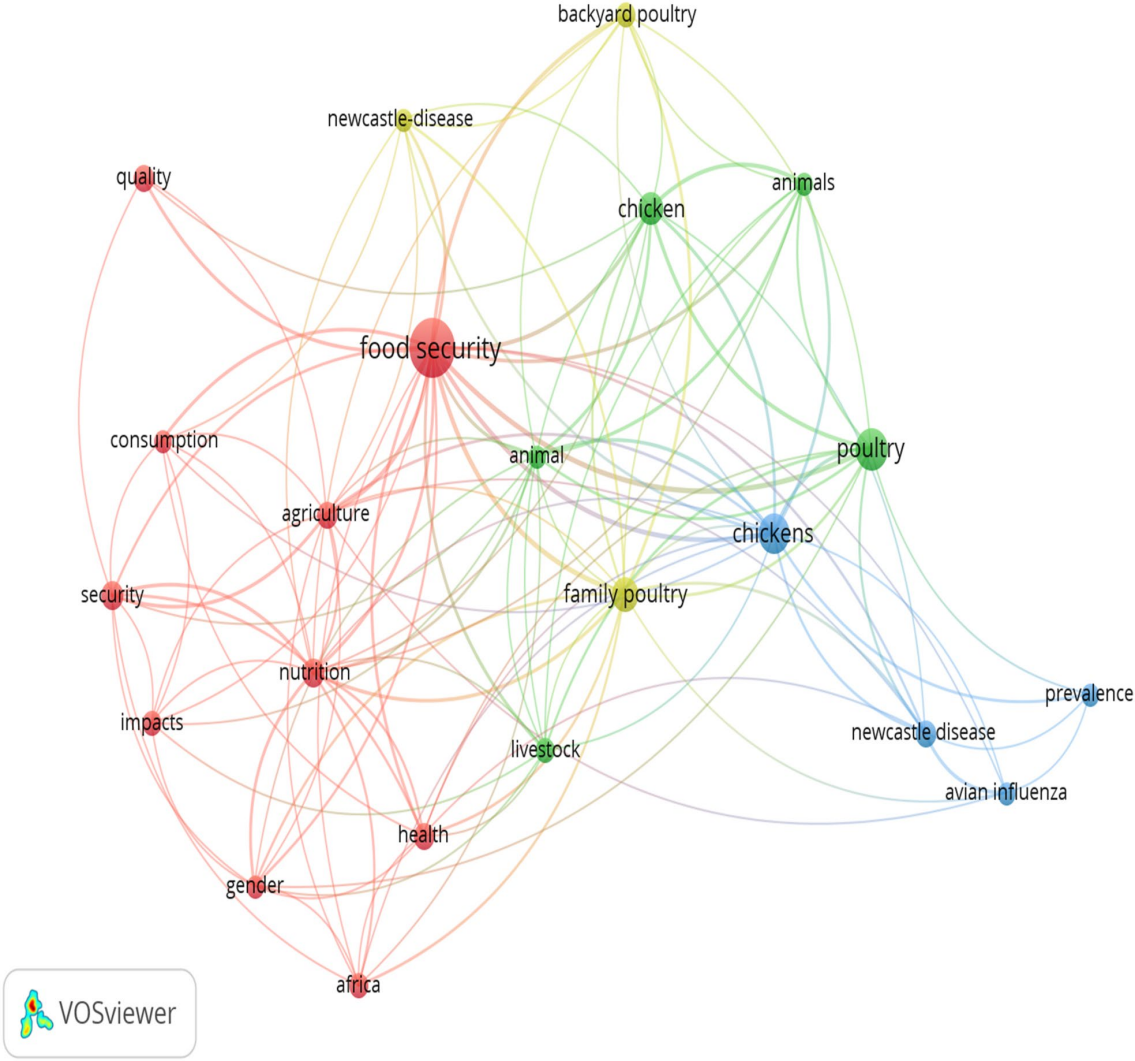
Keywords network visualization and association strength of global research on village chicken production and food security. Each node in the network represents individual keywords and the diameter of the node correlates with other keyword strengths. Lines/strokes represent the pathways of association between keywords

The result in Fig. 6 shows information about the keywords network visualization of commonly occurring keywords in research on village chicken production and food security. It is very essential to note that, each keyword node/size as seen in Fig. 6 depicts its strength and frequency in the literatures associated to village chicken production and food security research publications. Likewise, it can be inferred that the closer the keywords to each other, the more likely their interrelation in the literature during the study period. The network visualization of commonly occurring keywords simply depicts the common words in village chicken production and food security research which makes it stress-free to recognize areas of concentration in this field.

**Fig. 7:**
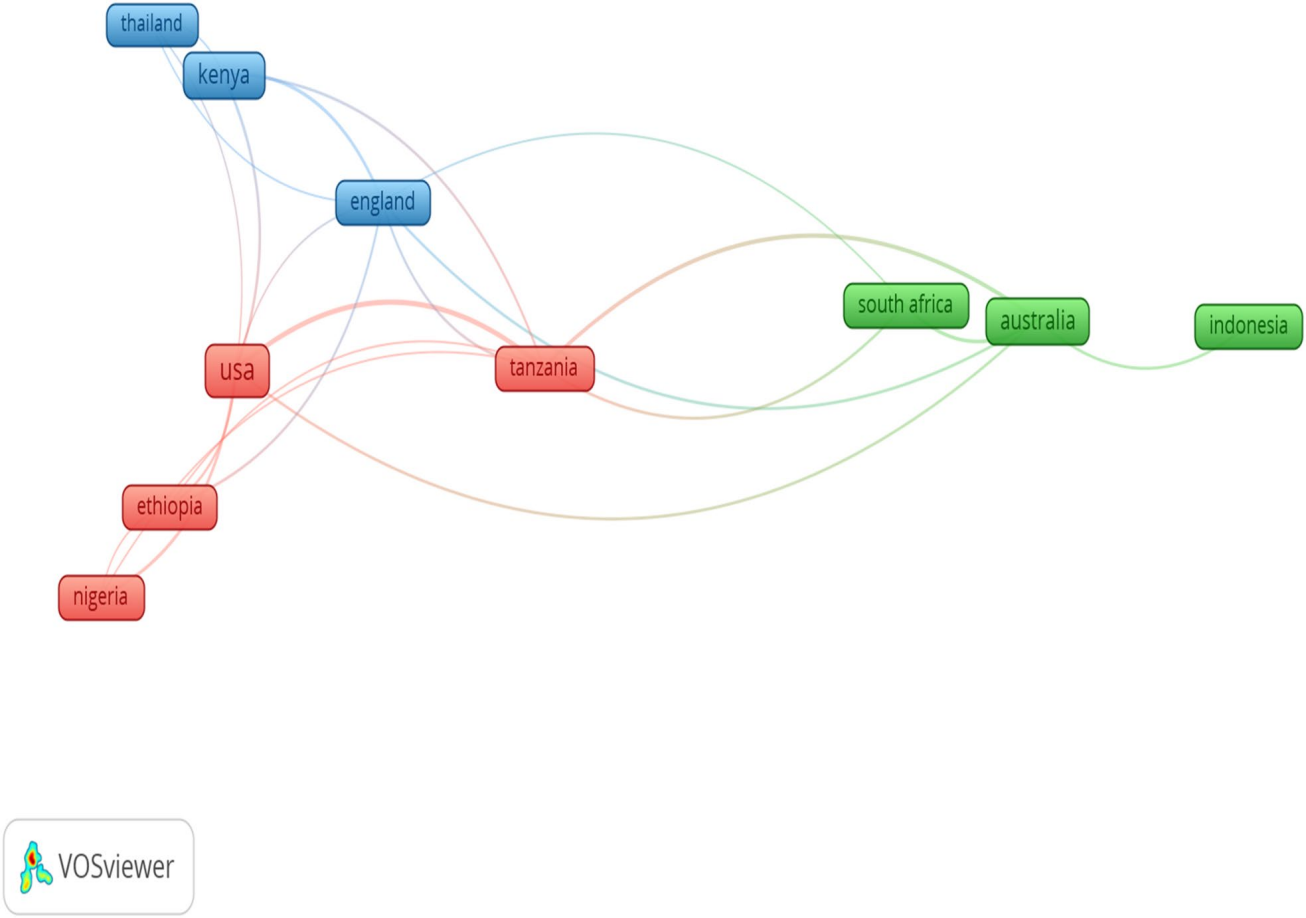
Collaborative mappings of networks of ten (10) countries on research done on village chicken and food security with at least five publications. Each node in the network is an individual country and the diameter of the node corresponds to the number of publications. The strokes denotes the paths of networking between countries and the thickness of strokes signifies the degree of collaboration between the countries, while the three (3) different colours seen in this figure represent the collaboration cluster of the countries

In addition, Fig. 7 shows network visualization map of countries’ collaboration, depicting 10 countries that had at least five publications. Each node in the network is an individual country and the diameter of the node corresponds to the number of publications by each country. The strokes denote the paths of networking between countries and the thickness of strokes signifies the degree of collaboration between the countries, while the 3 different colours (green, blue and red) represent the collaboration cluster of the countries. Collaboration links ranged from 2 to 18. Tanzania and USA both had the highest number of collaborations (*n* = 18, each), followed by Australia (*n* = 13), England (*n* = 12) and Kenya (*n* = 9), respectively. The top 11 most prolific research institutions with at least 6 research articles are listed in Table 8. The University of Sydney in Australia (number of articles = 19) was ranked first; Sokoine University of Agriculture in Tanzania and the Washington State University (Number of articles = 9) were ranked second and the University of Liverpool in the United Kingdom (Number of articles = 8) was ranked third position, respectively, among others.

**Table 8:** The topmost productive institutes on village chicken production linked with food security research with at least 6 article publications

S/N	Affiliations	Nations	Number of articles	Rank
1	University of Sydney	Australia	19	1st
2	Sokoine University of Agriculture	Tanzania	9	2nd
3	Washington State University	USA	9	2nd
4	University of Liverpool	United Kingdom	8	3rd
5	University of California, Davis	USA	7	4th
6	University of Florida	USA	7	4th
7	University of Queensland	Australia	7	4th
8	Cornell University	USA	6	5th
9	International Rural Poultry Centre	Australia	6	5th
10	University of Nairobi	Kenya	6	5th
11	University of Putra Malaysia	Malaysia	6	5th

## Discussion

The present study of village chicken and food security as a tool for reducing hunger was examined between 2000 and 2020 based on information obtained from WoS and Scopus data banks. It was noticed that the number of articles on village chicken production with respect to food security increased non-linearly from one (3) article in 2000 to 104 articles in 2020. However, our result showed fluctuations in article outputs on village chicken production and food security research from 2000 to 2016. This fluctuations, thereafter, experienced a stable rate of increase from 2017 (*n* = 10) to 2020 (*n* = 21) resulting into an annual rise of 12.93% of research articles. The observed increase in publication on village chicken and food security related articles suggests that more people in the field are now showing more interest in this research area. This growing interest may likely be due to the continual search for strategic and applicable approach to ameliorate global food security through the use of village chicken production. Several authors have reported the prospects of using village chicken as food source (meat and egg) for the poor (Akuili et al., 2007); [16], as improved nutritional enhancer [51], as source of income generation source [37], and as a means to improve the economic resilience of vulnerable people [16].

In addition, from the annual scientific production graph in Fig. 3 (with annual increase of 12.93%), there is a solid indication that more publications on the use of village chicken as food to reduce hunger and promote food security will further increase in the future. Although, judging from the aggregate numbers of articles from 2000 till date, it seems that the publications on village chicken production to boost food security are few. However, with the ever growing challenge of global hunger and food insecurity in many countries, and especially in developing countries [6], this may further drive the current generation of new scientific knowledge, towards a momentous increase in the amount of researches that will be done on this subject matter.

Unlike with other research areas, it was observed in the current study that many of the leading authors that are driving research on the use of village chicken production for food security are from developing countries and especially from Africa such as Nigeria, South Africa, Kenya, and Burkina Faso among other African countries. Other similar bibliometric studies that analysed researches from other scientific fields, usually report research/knowledge drivers from developed countries like the United States, the United Kingdom, China, Brazil, with few from very low-income countries [17, 42, 50].

The foremost countries with collaboration links on village chicken production and food security researches showed that some of the collaboration allied were among scientist from developed and high financially stable countries including United States, Australia and United Kingdom, while many others are from developing countries like Nigeria and Kenya among others whose nations economic status to funding research is still poor or very little. The United States have often shown dominance in several research fields as seen in several bibliometric studies [8, 25, 9]. How be it, our current observation appears to be in contrast with other bibliometric studies with regard to collaboration networks of countries as compared to other research fields that reported collaboration allied of their research from highly developed countries [42, 50]. Previous bibliometric studies showed that alliances between developed and developing countries are scare in several scientific fields [17, 42]. However, it should be noted that collaboration in scientific research from both intra- and international institutions between developing and developed countries could promote a more beneficial opportunities of pulling resources (funds and facilities) and more man-power in the case of division of labour to tackle vital research gaps in researches on village chicken production in relation to food security research. The economic strength of any nation is a contributing factor that motivates their research priority and performance [65] and [44]. The situation of food insecurity, hunger and poverty in most developing countries, and mostly in countries from sub-Sahara Africa should spark up more drive by the government and researchers in these countries to explore the prospect of doing more researches on the adoption of village chicken farming as a strategic tool for food security.

The low-input nature of backyard village chicken farming makes it accessible to these vulnerable people from several poor societies of many countries who are at a higher risk of food insecurity [15]. Village chickens can make important economical contributions to a small- or medium-scale families, both as a small means of steady income, or as a liquid asset, which can be utilized by these families to access good and healthy food thereby promoting financial security. According to Wong et al. [63], the economic contributions of village chicken production to households differ from place to place. Report has it that, village chicken production fetched an estimated amount of yearly income of thirteen (13) dollars to small families in Ethiopia [38]. Likewise, in other developing countries, the economic input of village chicken production to small vulnerable families, such as Haiti is 27 dollars [39], for Mozambique is 55 dollars [64] and 124 dollars in Nigeria [3]. According to Desta et al. [7], village chicken production could be a means of job opportunity for unemployed youths which in turn may have a long term effect of contributing to the political stability of some developing countries that are highly susceptible to political unrest due to high rate of unemployment among its citizens and especially the youths.

USA, Australia, Indonesia, Nigeria, South Africa and Kenya maintained top ranks among the 20 top nations that are in active research on the use of village chicken as tool to combat the challenge of food security in terms of aggregate amount of research publications (Table 3). One chief reason for any country to fall within the category of more number of article publications in a given research subject matter is well linked to the nations’ economic strength and access to research facilities and funding [30, 44, 68]. However, from a universal standpoint, the USA and Australia are seen to be the leaders in sustainable livelihood and in turn food security studies, in terms of quantity and quality of publications in this subject matter as well as in academic impact [67]. Again, the USA can be regarded as the most important partners in the network of international collaboration on sustainable livelihood [67]. The reason for this, is clearly that, they recognize early enough the importance of the concept of sustainable development and in turn food security with the use of available resource materials (including village chicken) for people globally, and keenly disseminate information relating to studies about it.

Conversely, result from the present study also indicate that some countries (e.g. Nigeria, Kenya, Indonesia, etc.) that had several research publications are from developing countries which gives an important picture of the subject matter in tackling food insecurity in these countries. Mottet and Tempio [34] in their study reported that village chicken contribute meaningfully to the production of meat and egg and these animal products were highly consumed in several low-income countries (including South Asia and sub-Saharan Africa). This further buttresses the importance of village chicken farming in promoting food security in these countries. Having the inclusion of some developing countries such as Indonesia, Nigeria, South Africa, and Kenya (among others) within the ranks of top article productions may reflect the challenging issues arising as a result of food insecurity faced in these countries.

There is a relatively low contribution to research outputs on village chicken production with regard to food security studies by developed countries especially from main Europe (with only Germany and United Kingdom making the list in the top 20 countries) as observed from the present study (Table 3). This observation may not be un-related to the fact that, researches of these nature (village chicken farming) may not find relevance (expect otherwise, if they want to collaborate in research with developing countries) in developed countries as they are well sustained in terms of food security and economic stability in most of these countries. In addition, these western European countries are also known to be largely involved in large-scale advanced poultry production as food for its citizens [52].

There were very few observed countries with multiple collaboration (MCP) on village chicken farming as related to food security research with USA having the highest incidence of multiple collaboration (*n* = 12) and Australia (*n* = 8), as seen in Table 3. Most countries in the list of top 20 most productive countries do not have collaborations (Table 3). This may not be unconnected to the fact that the present research field (village chicken production with regard to food security) seems new, but can be observed to be gradually gaining global recognition with an annual growth rate of 12.93% (Fig. 3). However, from our observation, it is expected and projected that more countries may likely get involved in multiple country collaborations judging from the current annual growth rate of research publications being generated in recent years in this field. Collaboration in scientific research from both intra- and international institutions between developing and developed countries will result in a more fruitful opportunities for pulling resources (funds and facilities) and more man-power for division of labour to tackle vital research gaps in the studies of village chicken production as related to food security research in the concerned countries.

There was position twist in the rankings among the top 20 countries who were considered as most active in the field of village chicken production as related to food security when research outputs was assessed using the criteria of total citation (TC) per country (Fig. 2 and Table 2). Our observation may not be new as similar observation has also been reported in other bibliometric studies [17, 42]. The reason for this position twist in rankings when using the total number of citations to judge nation or author’s publications may show its unreliability as a valid tool to measure productivity of countries in that regard. According to the claim by Fricke et al. [24], the rate of citation of a country does not really reflect publication outputs of that country or an author. The reason for this is because, the smaller the number of articles used for valuation in bibliometrics, the greater the impact of a few regularly cited articles [24]. In several cases, some authors have indulged in self-citations, while others give inaccurate citations when reporting their findings which in turn may produce false quality and quantitative metrics of total citations about a particular author or country [17].

The most commonly revealed keywords and research disciplines (including article outputs) related to village chicken production and food security study show the research hotspot during our survey period which include, food security, chicken, nutrition, animal, quality, livestock, family poultry, Newcastle disease, backyard poultry among others are all pointing to the direction of promoting researches and interventions that will meet/tackle the challenges of food insecurity that is bedevilling most individuals in some countries. Furthermore, the keywords and research discipline observed from our study disclosed some of the efforts made by authors to promote research activities on the use of village chickens as a possible tool for meeting the hunger needs of food in-secured people and to gain an understanding of the future prospect of the practical interventions/programmes for tackling food insecurity. This findings (most frequently occurring keywords) was further supported by other theoretical framework indexes such as the keywords network visualization diagram and the tree map (in Figs. 5 and  6). Overall, keywords tend to encapsulate the contents of a research publication, and it serve to concentrate and refine the principal concepts of the research [11].

The co-occurrence distribution gives an overview of research niche within the village chicken production and food security over a given period of time [46]. It adds to the grasping of the conceptual framework of the knowledge niche and mirrors the research network of scientists in the field [57]. It also unveils the nature of a research facade, recognizes developing trends; and highpoints prospective pivotal topics which is according to Freeman’s betweenness centrality [10, 55]. In Fig. 6 of our result, the size of nodes indicates the degree of the “betweenness centrality” of the nodes while the colour of the nodes is used to depict a cluster of co-cited journal references that are closely associated (the same colour belongs to the same cluster, and the theme is determined based on the clustering); village chicken and food security and the thicknesses of the strokes/lines linking the nodes denote the frequency of co-occurrence.

Among the top 20 journals that are relevant in researches on village chicken production and food security include Tropical Animal Health and Production and Food Security journals with H_index of 4 and 3, respectively. These journals are well known in promoting research studies related to livestock and food production with respect to improving food production and security [1, 14, 16]. More journals should be encouraged to make calls like the United Nations and the Food and Agricultural Organization [22, 59] on topical issues and researches related to promoting studies that will enhance livestock production and food security due to the global threat/challenges of hunger and poverty by vulnerable people.

Of essence to note from a specific point of view, is that, in the study of one of the top 20 most cited articles by Dumas et al. [16], it was reported that, improved intervention programmes (including Newcastle disease vaccination and good housing and management system) boost village chicken poultry as a practical measure to promote food security, social, agricultural, and ecological resilience among financially constraint people. From the intervention program reported by Dumas et al. [16], it was shown that the resilience of the backyard village poultry production system was significantly improved via Newcastle disease vaccination and simple housing managements, and this improvement was visibly noticed in the rise of the average flock size of village chickens recorded in the local households. Some of the highlights of the study by Dumas et al. [16] who reported the prospect of improved village chicken production among local financially constrained people include:

- It helped generated increase in family revenue.
- It boosted food security due to improved access to eggs and meat from village chicken.
- Assisted households in diversifying income generated from sales of surplus return from meat and eggs, making them resilient to any sudden external or internal shocks that may surface in the family.

The study by Dumas et al. [16] offers an inclusive and vibrant intervention and policy approach and a structure for systematic adaptation and evaluation on the current subject matter. Village chicken production can also be used to improve food security in some indirect ways as explained by Wong et al. [63],such as improving nutrient usage and recycling in the environment (manure from chickens), adding value to mixed farming practices, contributing to women’s empowerment, and facilitating admittance to healthcare and education, contribute to some of the Sustainable Development Goals (SDG), and to future food security through supporting biodiverse genomes.

The phrases ‘international papers’ or ‘international co-publication’ is often used to select manuscripts that are published via the cooperation of researchers from different countries [26]. Our result in Fig. 7 demonstrates international cooperation ties for 10 selected countries that are involved in village chicken production and food security research. Representing nations as the nodes within the network (the size of each node representing the sum of published articles), strokes/lines between the nodes represent collaboration between collaborating nations (the thickness of a strokes/line indicating the number of international co-published articles or findings). From a universal viewpoint, it is remarkable that the foremost research nations may have formed a close and united collaboration network on research areas that is of interest among them [67]. Nations of the world derives satisfaction to work collaboratively chiefly in their strong subject areas in the field of knowledge sharing and science. Research collaboration among nations undeniably has a positive and significant impact on published outputs [27, 61]. As noticed from our result, the United States of America had academic institutions and organizations that had the major co-author associates in international co-authorship in the field of village chicken production and food security research, which was followed by Tanzania, Australia and UK (Fig. 7). Similar observation to our findings has also reported the USA as a foremost academic destination for collaboration and co-authorship in other research fields [17, 67].

Overall, more research on the use of village chicken to tackle the challenge of food security is worth investigating. A bibliometric study couple with a meta-analysis or a narrative review in village chicken production and food security research may also be of great importance to the collection of scientific knowledge in this field.

To date, our paper appears to be the first bibliometric study that reported the outputs of peer-reviewed articles on village chicken production as related to food security at a global level. However, we are aware that there might be some short-comings to the present study which may include and not limited to:

i. Missing research outputs that we might not have retrieved in the analysis of village chicken production and food security research or its related words during the retrieval of data from the two used data banks, namely: WOS and Scopus.
ii. Short-comings may likewise arise from this study since we did not add publications on village chicken production as related to food security research that were in non-indexed journals and thus, would not have been available in WOS and Scopus databanks, such as the ones published in some non-English journals.
iii. The present study may also be limited due to the exclusions of other article types including conference abstracts, and note papers, etc.

## Conclusion

The present bibliometric analysis revealed a steady increase in the use of village chickens production as a potential tool to boost food security, with greater research outputs from low- and middle-income when compared to high-income nations and limited collaboration with developing nations based on the WOS and Scopus data banks. The high research publications in several developing countries on the current subject matter mirrored the fact that more developing countries are the ones facing challenges relating to food security and its related issues like poverty and lack of adequate quality nutrition in the diets of vulnerable and financially constrained people. Regardless of the substantial progress illustrating the utilization of village chicken production as tool for food security and hunger alleviation over the past 20 years, many questions remained to fully address their (village chicken production) sustainability as a standard measure/practice to tackle the multi-dimensional challenges of food security and hunger alleviation in countries of the world and especially developing countries.

### Future perspectives of the use of village chicken production to boost food security

The production of village chicken can be improved meaningfully with suitable changes to management and best practices to increase chicken production for food. There is so much wealth of knowledge, and practice related to village chicken production, which together with recognized local and cost-effective interventions incorporating improvements in the production of village chickens such as nutrition (from domestic and environmental waste), housing and/or management, and/or genetics that have the prospect to achieve sustainable production of indigenous chickens for the benefit of vulnerable and food insecure people.

For instance, the multipurpose (meat and egg) village chickens can be employed to sustain the livelihood of people that are at risk of food insecurity across low- and middle-income countries; although, the potential impact of village chicken production to boost food security is often overlooked. One of the ways village chicken production can be improved in a sustainable farming system is by integrating it with mixed crop–livestock farming system that is practiced in developing nations. This system of farming integration augments the efficient use of scare available resources and allows for the provision of multiple products (eggs, meat, offals, manures, and feather, etc.) from village chickens. Although, it could be noted that there is no known standardized management system in raising village chickens; consequently, there is an extensive pool of local/traditional knowledge, and practice in raising them which may vary from country to country. The indigenous acquired wisdom and knowledge serves as an essential resource to invent and to recognize suitable interventions for village chicken production if it will be employed to improve food security in developing countries. This intervention/s, however, may necessitate an exhaustive study of the local management farming practice system. To expedite a high adoption of new technologies in village chicken production, cost-effective interventions is required to be appraised in the application of these farming practices in the local context. Finally, with suitable support from all stakeholders including government, research institutes and community organizations, improved interventions and adoption of innovative village chicken production has the potential to meaningfully and sustainably enhance the livelihoods of vulnerable and financially constrained people in developing countries.

## Data Availability

Will be made available on request.
